# Endodontic Surgery Management of a Large Periradicular Actinomycosis Lesion

**DOI:** 10.1155/crid/3573454

**Published:** 2025-11-10

**Authors:** Rahhali Mohamed, Belhaj Khalifa, Sakout Majid

**Affiliations:** ^1^Conservative Dentistry and Endodontics, Faculty of Dentistry, Mohammed V University, Rabat, Morocco; ^2^Oral Surgery, Faculty of Dentistry, Mohammed V University, Rabat, Morocco

**Keywords:** actinomycosis, apical periodontitis, endodontic surgery

## Abstract

A 22-year-old female patient consulted the Odontology Department of the Mohammed V Military Hospital in Rabat, Morocco, for a recurrent abscess in relation to the left maxillary central incisor (#21). The dental history of the tooth began with necrosis following trauma a few years before. A conventional endodontic root canal treatment was performed, but several infectious episodes occurred, treated only with antibiotics. The radiographic examination showed an adequate root filling and a large apical lesion in Tooth #21. Tooth #21 was diagnosed as chronic apical periodontitis. After orthograde retreatment, clinical signs have not resolved, and the patient has consulted for a recurrent abscess. Given this atypical clinical situation, an extraradicular infection was suspected and the decision for endodontic surgery was made. The histopathological diagnosis revealed actinomycotic infection. The aim of this case report is to draw practitioners' attention to this rare infectious etiology, which does not respond to conventional endodontic treatment, and to expose the diagnostic and therapeutic approach required to treat apical actinomycosis.

## 1. Introduction

Surgical endodontics is a retrograde endodontic treatment indicated after the failure of initial endodontic therapy, and possibly after an attempt at orthograde retreatment. Thanks to recent advances in endodontics, particularly improved imaging technologies, refined microsurgical instruments, and the introduction of bioactive root-end filling materials [1], surgical endodontics has become a predictable and reproducible procedure, with success rates approaching 90%. This greatly enhances long-term tooth retention [2].

Residual endodontic infection or secondary infection due to coronal leakage is a common cause of treatment failure. However, less frequent and often overlooked etiologies such as extraradicular actinomycosis may also be involved [3].

Actinomycosis is increasingly recognized as a critical factor in persistent periapical lesions and the failure of conventional endodontic treatments [4, 5]. Unlike typical intraradicular infections, *Actinomyces* species can establish extraradicular colonies within periapical tissues, forming dense, granulomatous masses that are encapsulated and thus isolated from the root canal system [4]. These colonies are resistant to standard irrigation protocols and may remain asymptomatic for prolonged periods, only to present later as chronic, nonhealing periapical pathologies. As a result, they often evade both diagnosis and treatment unless surgical intervention is undertaken [6]. The definitive diagnosis requires histopathological analysis or extended anaerobic culture, and treatment typically involves both surgical excision and prolonged antibiotic therapy [5]. Failure to identify and manage such infections appropriately can result in repeated endodontic failures despite technically adequate procedures [7].

This clinical case aims to highlight the importance of considering actinomycosis in the differential diagnosis of persistent periapical pathology and to present the therapeutic approach required for successful management.

### 1.1. Clinical Case

**Figure 1:**
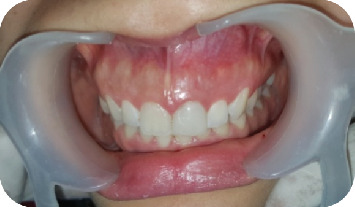
Clinical examination showed tumefaction in relation to Teeth 21 and 22.

**Figure 2:**
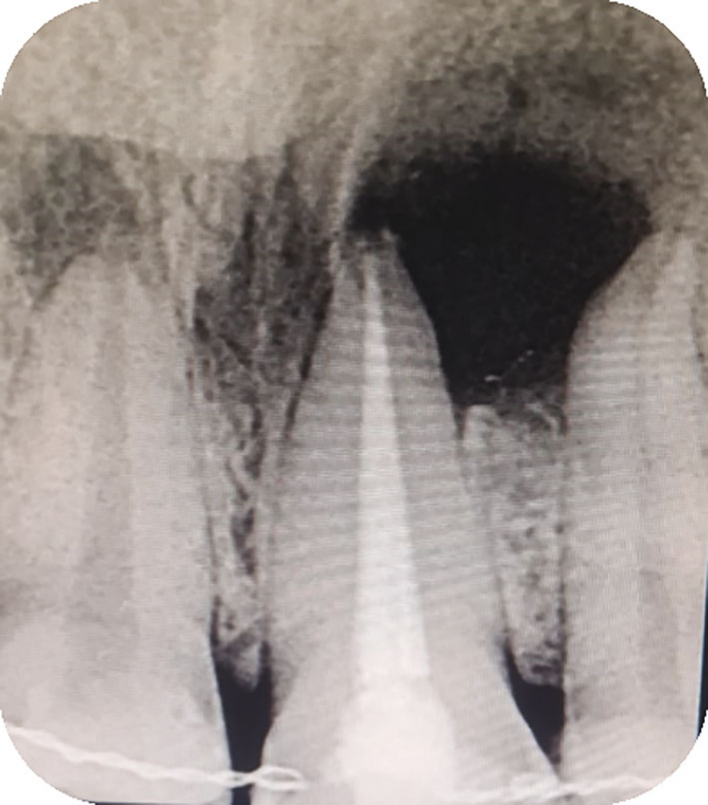
Radiographic examination showed correct root canal filling of Tooth 21.

**Figure 3:**
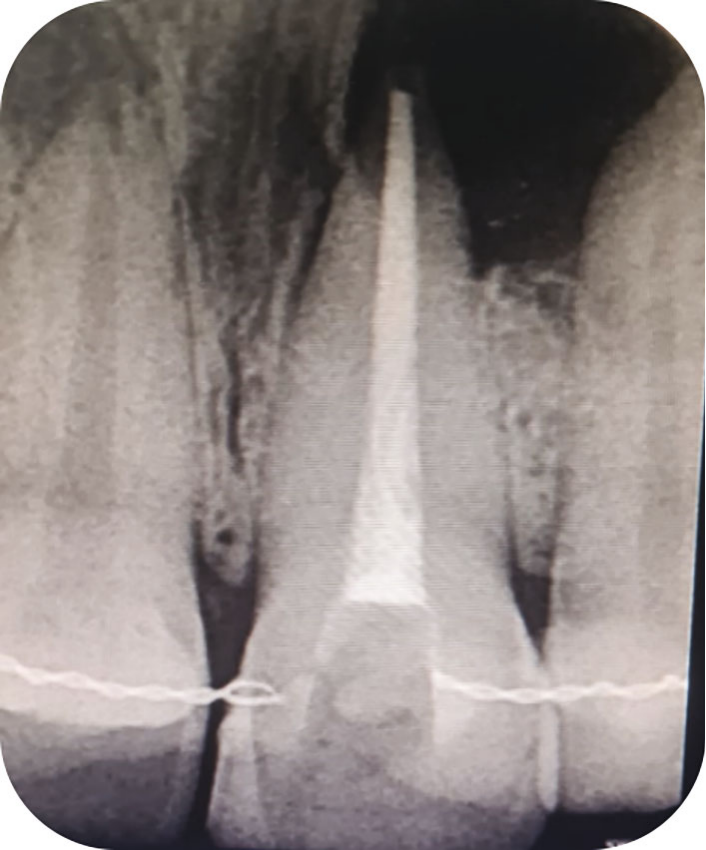
Radiographic showed root canal obturation after root canal retreatment.

**Figure 4:**
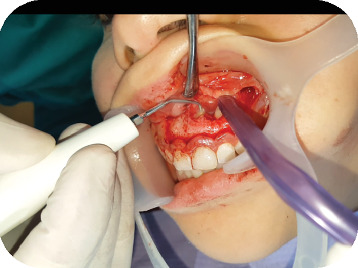
Retrograde preparation with ultrasonic insert Acteon AS6D.

A 22-year-old female presented to the Odontology Department of Mohammed V Military Hospital in Rabat, Morocco, with a recurrent abscess related to the left maxillary central incisor (#21). The patient had no systemic diseases, drug, or food allergies. The dental history of the tooth began with necrosis following trauma a few years prior, which was treated with conventional root canal therapy. However, the treatment relapsed, with multiple infectious episodes managed solely by antibiotics. The extraoral clinical examination revealed no abnormalities. Intraorally, an adequate restoration was observed on Tooth #21, with a firm and slightly tender swelling noted around Teeth #21 and #22 (Figure 1), normal periodontal probing depths, and slight tenderness upon percussion of Tooth #21. A positive response to cold testing was observed in the left maxillary lateral incisor (#22) and the right maxillary central incisor (#11), indicating that these teeth were vital and unrelated to the recurring abscesses. Radiographic examination revealed adequate canal obturation and a large apical lesion associated with Tooth #21 (Figure 2). The diagnosis of chronic apical periodontitis was made for Tooth #21. Orthograde endodontic retreatment was performed (Figure 3), but the clinical symptoms persisted, and the patient returned with a recurrent abscess within 3 months. Given this clinical scenario, an extraradicular infectious cause was suspected, which may have evaded the effectiveness of conventional treatments, and surgical management was decided upon. Following enucleation of the cystic lesion, a 2-mm apical resection of the root was performed. Retrograde preparation was completed using an ultrasonic tip under constant irrigation with saline solution (Figure 4). After achieving hemostasis, the endodontic system was sealed off from the deep periodontium with a retrograde Biodentine filling. Medical treatment included amoxicillin (1 g, twice daily, orally for 4 weeks) following surgical excision of the lesion, which is considered sufficient due to the relatively circumscribed nature of apical actinomycosis.

**Figure 5:**
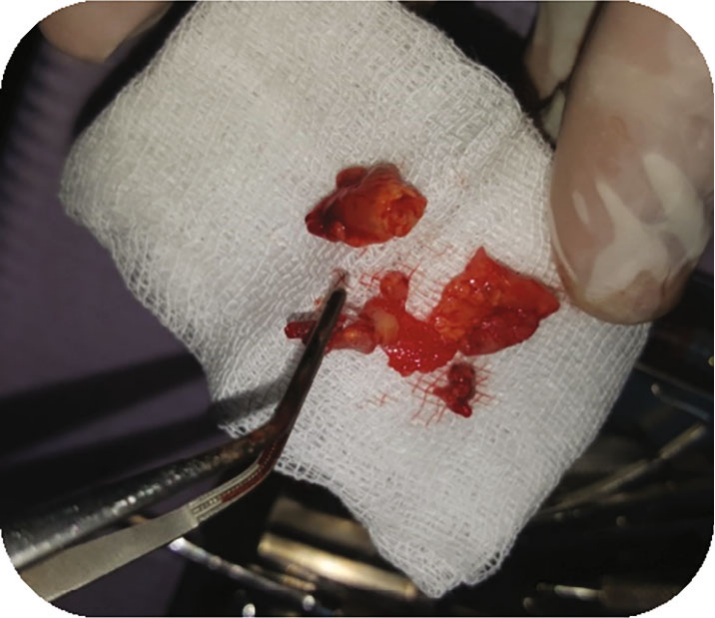
The biopsy specimen showed a sulfur granule-like structure.

**Figure 6:**
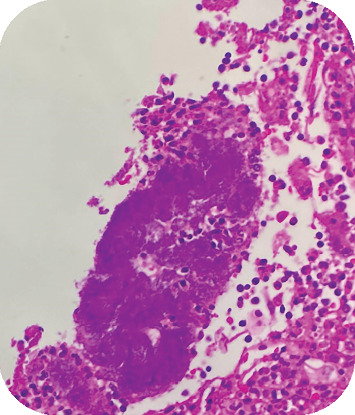
Pathological examination shows the content of a radicular cyst with nonspore-forming, filamentous rod bacterial colonies, with radiating, club-shaped filaments.

The histological specimen was sent to the pathology laboratory, where the lesion showed inflammatory cells and nonsporulating Gram-positive filamentous rods, with areas of suppuration containing sulfur granules (Figure 5), indicating a histopathological diagnosis of an apical actinomycotic lesion (Figure 6).

**Figure 7:**
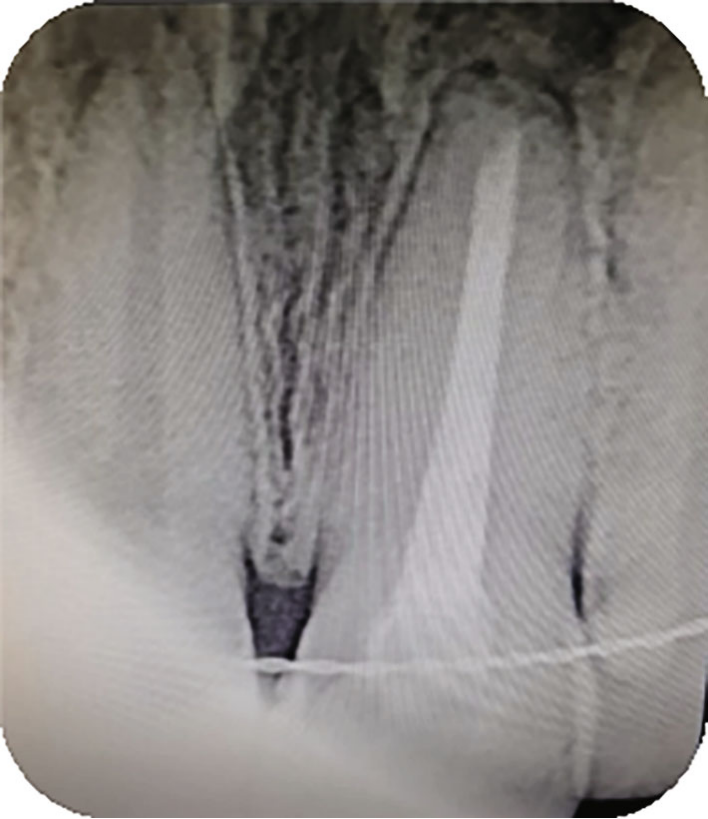
Radiographic examination 12 months after apical surgery.

Clinical and radiographic follow-up was initiated at regular intervals, during which the resolution of the infectious signs was noted, and the tooth became asymptomatic. Radiographically, significant apical bone healing was observed at 12 months, with the reappearance of the apical periodontal space and lamina dura, indicating good periodontal health (Figure 7).

## 2. Discussion

Endodontic treatment failure is generally linked to inadequate disinfection of the endodontic system, with the persistence of a residual bacterial load exceeding a critical threshold, which maintains an inflammatory state in the periapical tissues. In most cases, initial endodontic treatment failure can be managed by orthograde endodontic retreatment. However, in certain clinical situations, this may be insufficient, requiring endodontic surgery or retrograde endodontic treatment [3].

Endodontic surgery is typically indicated after the failure of orthograde retreatment, but it may also be indicated for prosthetic reasons, such as complex root-anchored prosthetic restorations, where removal is difficult or risky [8]. Endodontic treatment failure may also be due to extraradicular causes, for which even technically correct orthograde canal treatment remains insufficient. These causes include inflammatory reactions to exogenous substances such as cellulose fibers from paper points or debris from coronal filling materials, true cysts that do not communicate with the tooth's endodontic system, and cholesterol crystals [3].

Extraradicular infection caused by *Actinomyces propionicus* can also be a factor [6, 7]. This microorganism is part of the commensal flora of the oral cavity and can be found in dental carious lesions and in the biofilm of periodontal pockets. Its prevalence in the total oral flora is approximately 97% within the first 2 years of life [6]. These bacteria become pathogenic when they breach mucosal barriers due to infection or trauma, as in the clinical case presented. Apical actinomycotic infection is a rare condition that can explain the failure of conventional endodontic treatments [9], due to the organization of these bacteria into a filamentous biofilm in cemental crypts [6]. This extraradicular disposition protects the bacterial biofilm from the antibacterial measures employed [7].

Actinomycotic infection is characterized by recurrent abscesses and contains granules known as “sulfur granules,” which are tangled masses of branching *Actinomyces* filaments. Fibrous zones separate areas of suppuration, acting as a barrier that isolates the infection and significantly reduces the efficacy of endodontic disinfection. This infection can be dependent or independent of intraradicular infection [10].

Although the incidence of apical actinomycosis is rare and relatively low, it should be considered in cases of orthograde endodontic retreatment failure, especially when associated with recurrent abscesses. Treatment of apical actinomycotic infection requires a combination of surgical excision of the pathological tissue and antibiotic therapy based on penicillin, as indicated in previous studies [11]. Dentists should be aware of this cause of endodontic treatment failure, despite its rarity.

## Data Availability

The articles used in this article are listed in the bibliography. If necessary, the corresponding author can share these articles with you.
